# DNA Methylation of Mouse Testes, Cardiac and Lung Tissue During Long-Term Microgravity Simulation

**DOI:** 10.1038/s41598-019-44468-w

**Published:** 2019-05-28

**Authors:** Sergey S. Loktev, Irina V. Ogneva

**Affiliations:** 1 Cell Biophysics Lab, State Scientific Center of the Russian Federation Institute of Biomedical Problems of the Russian Academy of Sciences, Khoroshevskoyoe shosse, 76a, Moscow, 123007 Russia; 2 I. M. Sechenov First Moscow State Medical University, 8-2 Trubetskaya St., Moscow, 119991 Russia

**Keywords:** Cell signalling, Cytoskeleton

## Abstract

Under microgravity, the gene expression levels vary in different types of cells; however, the reasons for this have not been sufficiently studied. The aim of this work was to evaluate the methylation of CpG islands in the promoter regions of the genes encoding some cytoskeletal proteins, the total methylation and 5 hmC levels, and the levels of enzymes that regulate these processes in the testes, heart, and lungs in mice after a 30-day microgravity modeling by antiorthostatic suspension and after a subsequent 12-hour recovery as well as in the corresponding control group and identical groups treated with essential phospholipids. The obtained results indicate that under modeling microgravity in the examined tissues a decrease of cytoskeletal gene expression (mainly in the heart and lungs tissues) correlated with an increase in the CpG islands methylation and an increase of the expression (mainly in the testes tissue) – with a decrease of the CpG-methylation, despite of the fact that in the examined tissues took place a decrease of the content methylases and demethylases. But the deacetylase HDAC1 content increased in the heart and lungs tissues and decreased in the testes, letting us suggest its participation in the regulation of the methylation level under microgravity conditions.

## Introduction

To date, the mechanisms by which the gravitational field interacts with living systems, particularly at the cellular-molecular level, remain unclear. For example, it is unclear how the long-term and transient changes in the expression of a number of genes occur^1–9^.

In eukaryotes, the DNA in cells exists as a chromatin, a complex with a number of proteins, which means that there exists a number of ways to regulate its expression. Thus, post-translational modifications of histones (acetylation, phosphorylation, methylation, sumoylation, etc.) or chromatin remodeling that changes nucleosomal organization (for example, with the SNF2H and SWI/SNF family complexes) may take place. In addition, the most important regulator of protein product formation is RNA interference, and DNA itself may also be modified using cytosine methylation in CpG islands. This last modification is a widely used mechanism for the regulation of gene expression in higher eukaryotes. In the genome of somatic cells in mammals, almost 70% of all CpG sites are methylated^10^; however, these sites these are not in the promoter regions^11^, which leaves the opportunity for regulation^12^ and explains why, for higher eukaryotes, DNA methylation is one of the key epigenetic factors^13,14^. However, under space flight conditions, data on this issue are scarce. Rice germs have shown hypermethylation, which did not correlate with the level of gene expression^15^, although Singh *et al*.^16^ demonstrated DNA hypomethylation using a model system with human T lymphocytes. At the same time, changes in the levels of not only methylation but also hydroxymethylation were observed in lymphoblastoid cells^17^.

Our data, which was obtained from the study of murine heart and lung tissue samples that were collected under space flight conditions, indicate that the total level of DNA methylation increases by 21–32% under space flight conditions, while the expression levels of methylase/demethylase genes do not change, except for demethylase Tet2 (hydroxylase), which decreases^9^. Although TET hydroxylases may participate in 5-hydroxymethylcytosine (5 mC) conversion to 5 hmC, the role of 5 hmC is still unclear. 5 hmC is suggested to be an intermediate product before a completely unmethylated state, but its role in regulating expression is not fully understood^18^.

Our previous studies show that oral administration of phospholipids, which have unsaturated fatty acids in their tails, can prevent the change in the pattern of expression of genes encoding cytoskeletal proteins in various cell types under the conditions of microgravity simulation^8^. Therefore, in this study, we decided to compare the pattern of expression of genes that encode cytoskeletal proteins and the methylation of CpG islands in their promoter sites with and without the administration of essential phospholipids. In addition, we assessed the overall level of methylation, the content of 5-hydroxymethylcytosine and the levels of enzymes ensuring the establishment/maintenance of the appropriate level of methylation.

## Results

### Body and tissues weight

There were no differences in the weights of the animals across all groups (Table 1). Compared to the weight of the testes in the C group, the weight of the testes was lower in the suspension groups and did not recover after 12 hours of recovery. Compared with the weight of the testes in the C group, the weight of the testes was 27% less in the 30 HS group (p < 0.05), 32% less in the 30 HS + 12 h group (p < 0.05), 34% less in the 30 HSE group (p < 0.05), and 36% less in the 30 HSE + 12 h group (p < 0.05).

**Table 1:** Mass parameters.

Parameter Group	Body mass, g	Testis mass, mg	Heart mass, mg	Lungs mass, mg
C	28.2 ± 0.7	259 ± 20	161 ± 11	212 ± 9
30 HS	27.0 ± 0.9	188 ± 18*↓	255 ± 13*↑	230 ± 18
30 HS + 12 h	26.1 ± 0.7	177 ± 16*↓	146 ± 6	182 ± 7*↓
CE	28.4 ± 0.5	237 ± 23	149 ± 5	224 ± 9
30 HSE	25.0 ± 0.9	171 ± 21*↓	221 ± 25*↑	220 ± 12
30 HSE + 12 h	26.0 ± 0.8	165 ± 15*↓	143 ± 6	178 ± 9*↓

*p < 0.05 in comparison with control group C.

Unlike the weight of the testes, compared to the weight of the heart in the C group, the weight of the heart in the groups with suspension was higher; specifically, it had increased by 58% (p < 0.05) in the 30 HS group and by 37% (p < 0.05) in the 30 HSE group compared to the weight of the heart in the C group. However, after 12 hours of recovery, in both the 30 HS + 12 h group and in the group with essential phospholipids, 30 HSE + 12 h, the weight of the heart did not differ from that in the C group.

The weight of the lungs did not change as a result of antiorthostatic suspension, but decreased after 12 hours recovery; compared to the weight of the lungs in the C group, that in the 30 HS + 12 h group was 14% (p < 0.05) lower and that in the 30 HSE + 12 h group was 16% (p < 0.05) lower.

### Relative mRNA level of genes encoding cytoskeletal proteins and the level of methylation of CpG islands in their promoter regions

#### Testes

Under conditions of microgravity, in the testes, no changes or increase were observed in the relative mRNA levels of genes encoding some cytoskeletal proteins, while there was a decrease in the level of methylation of CpG islands in their promoter regions.

**Figure 1:**
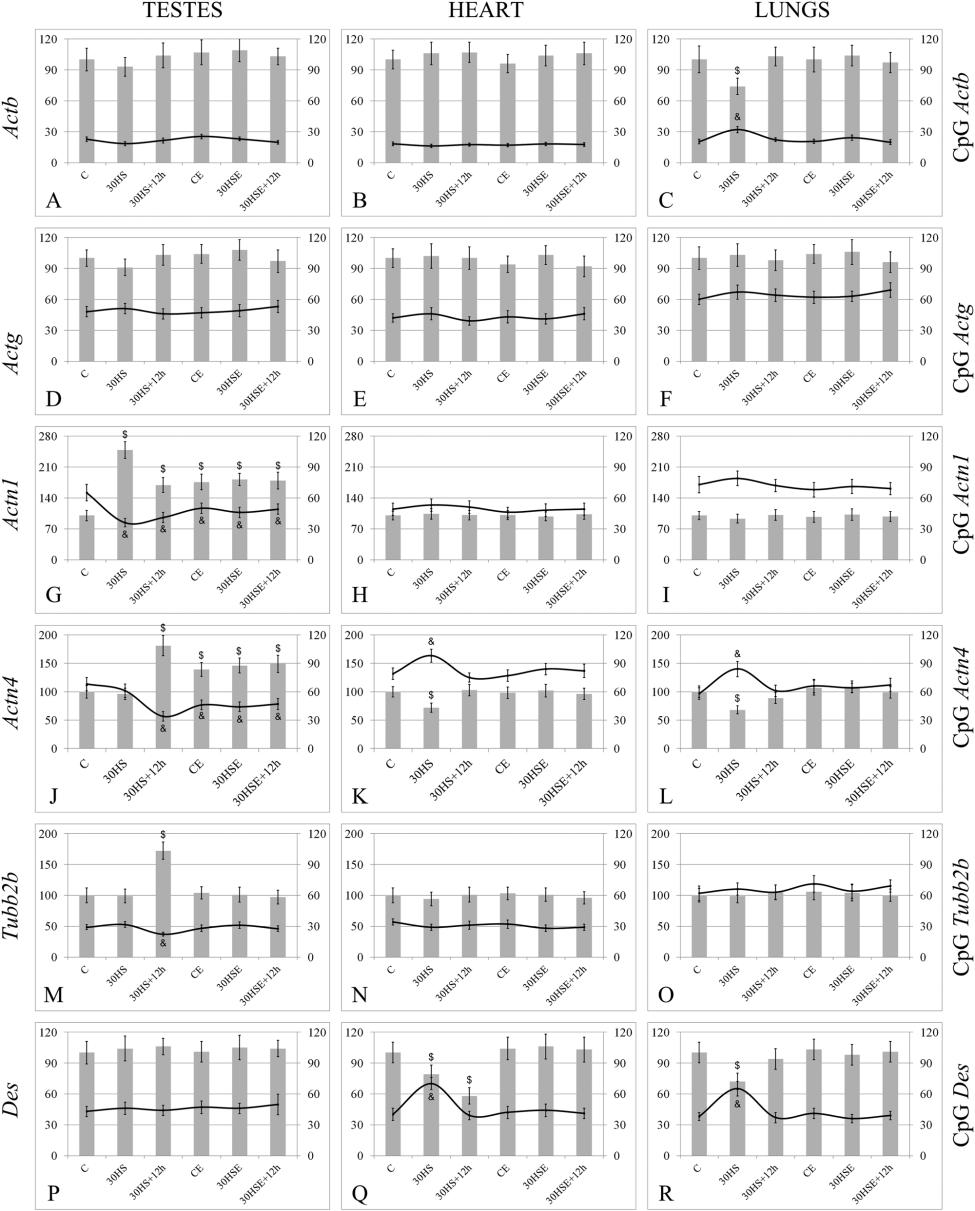
Relative mRNA levels of genes encoding cytoskeletal proteins and the methylation levels of the CpG islands in their promoter regions. ^$^p < 0.05 in comparison with group C, was used for significant differences in the relative mRNA level; ^&^p < 0.05 in comparison with group C, was used for significant differences in the CpG methylation levels. The left hand y-axis shows relative mRNA level and was used for the histogram columns, while the right hand y-axes shows the CpG methylation level in the promoter region of the corresponding gene and was used for the line graph. (**A–C**) *Actb* gene (testes, heart and lungs); (**D–F**) *Actg* gene (testes, heart and lungs); (**G–I**) *Actn1* gene (testes, heart and lungs); (**J–L**) *Actn4* gene (testes, heart and lungs); (**M–O**) *Tubb2b* gene (testes, heart and lungs); (**P–R**) *Des* gene (testes, heart and lungs).

Thus, the mRNA levels of beta-actin (Fig. 1A), gamma-actin (Fig. 1D), and desmin (Fig. 1P) did not change in any of the study groups.

The relative mRNA level of the *Actn1* gene was 148% (p < 0.05) higher in the 30 HS group than in the C group, and 12 hours of recovery appeared to decrease the level, although it did not return to the level of the C group; the mRNA level of the *Actn1* gene in the 30 HS + 12 h group was 69% than that in the C group (p < 0.05) (Fig. 1G). At the same time, the level of CpG island methylation was significantly lower in the 30 HS and 30 HS + 12 h groups than in the C group. In the groups administered essential phospholipids, suspension and subsequent recovery did not lead to changes in the mRNA level of the *Actn1* gene (CE, 30 HSE, 30 HSE + 12 h groups had no significant differences), but these levels were 76–82% higher than the level in the C group (p < 0.05), with decreases in the methylation levels of the corresponding CpG islands.

The relative *Actn4* gene mRNA levels were not different in the 30 HS group and the C group, but unlike *Actn1*, the *Actn4* mRNA level was 72% (p < 0.05) higher in the 30 HS + 12 h group than in the C group (Fig. 1J). However, the use of essential phospholipids led to the same effect seen for *Actn1*; there were no significant differences among the CE, 30 HSE, 30 HSE + 12 h groups, but the *Actn4* mRNA levels in those groups exceeded that in the C group by 39–49% (p < 0.05), while decreases were observed in the level of CpG methylation in the corresponding promoter regions.

For the gene encoding beta-tubulin, the changes in the relative level of mRNA were observed only in the 30 HS + 12 h group (Fig. 1M), where the level was 72% (p < 0.05) higher than that in the C group, with a corresponding decrease in the level of methylation of the CpG islands in the beta-tubulin promoter region.

#### Heart

For the heart tissue, no changes were observed in the expression of genes encoding beta-actin (Fig. 1B), gamma-actin (Fig. 1E), alpha-actinin-1 (Fig. 1H), and beta-tubulin (Fig. 1N), neither were there any changes in the methylation levels of the investigated CpG-islands in the corresponding promoter regions.

Unlike in the testes, compared with the C group, the 30 HS group had a 28% (p < 0.05) lower mRNA level of the alpha-actinin-4 encoding gene (Fig. 1K), which correlated with the increase in CpG islands methylation observed in its promoter region. The *Actn4* mRNA levels were not different between the 30 HS + 12 h and C groups.

The relative mRNA level of the gene encoding desmin (Fig. 1Q) was 21% (p < 0.05) lower in the 30 HS group than in the C group, while the level of methylation of CpG islands in its promoter region correspondingly increased. However, the *Des* mRNA level in the 30 HS + 12 h group was only 58% (p < 0.05) of that in the C group, while the CpG methylation level decreased relative to that in the 30 HS group and did not differ from the control.

The use of essential phospholipids led to no changes in the levels of expression of the investigated genes or in the methylation of the CpG islands in their promoter regions.

#### Lungs

In the lung tissue, the relative mRNA levels of the genes encoding beta-actin (Fig. 1C), alpha-actinin-4 (Fig. 1L), and desmin (Fig. 1R) were lower in 30 HS group than in the C group by 26% (p < 0.05), 32% (p < 0.05), and 28% (p < 0.05), respectively, which correlated with increases in the methylation of CpG islands in their promoter regions. At the same time, no such changes were noted when essential phospholipids were administered.

No changes were observed in the mRNA or methylation levels of the promoters of *Actg* (Fig. 1F), *Actn1* (Fig. 1I), and *Tubb2b* (Fig. 1O) genes among the C, 30 HS, and 30 HS + 12 h groups.

### The total level of methylation and the relative level of 5 hmC

**Figure 2:**
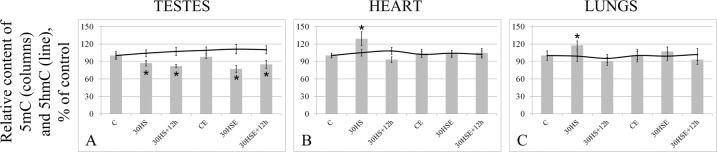
Total methylation and 5 hmC levels. *p < 0.05 in comparison with group C, was used for significant differences in the total methylation level. Histogram columns were used for the total methylation level, while the line graphs represent the level of 5 hmC. (**A**) testes, (**B**) heart, (**C**) lungs.

The total level of methylation in the testis tissue (Fig. 2A) was 13% lower in the 30 HS group (p < 0.05) and 23% lower in the 30 HSE group (p < 0.05) than that in the C group, and did not recover after 12 hours, remaining 18% (p < 0.05) lower in the 30 HS + 12 h group and 15% (p < 0.05) lower in the 30 HSE + 12 h group compared to that in the C group.

In the heart (Fig. 2B) and lung (Fig. 2C) tissue, the total methylation level changed only in the 30 HS group, in which it was higher by 29% (p < 0.05) and 18% (p < 0.05), respectively, compared to the levels in the C group.

No changes in the relative content of 5 hmC were observed in any of the tissues studied.

### The relative levels of methylases/demethylases and acetylases/deacetylases

#### Testes

**Figure 3:**
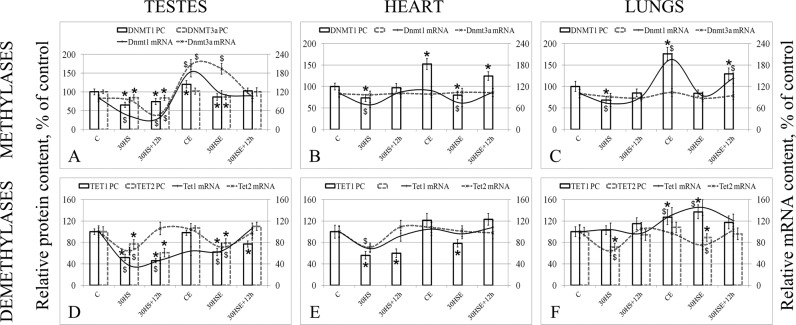
Relative methylation/demethylation fermentas protein and mRNA levels. *p < 0.05 in comparison with group C, was used for significant differences in the relative protein content; ^$^p < 0.05 in comparison with group C, was used for significant differences in the relative mRNA content. The left hand y-axis shows the relative protein level and was used for the histogram columns, while the right hand y-axis shows the relative mRNA level and was used for the line graph. (**A–C**) methylases DNMT1 and DNMT3a (testes, heart and lungs respectively); (**D–F**) demethylases of the TET-family, TET1 and TET2 (testes, heart and lungs respectively).

In the testes, compared to the level in the control group, the relative level of S-phase methylase DNMT1 (Fig. 3A) was 35% (p < 0.05) lower in the 30 HS group and 14% (p < 0.05) lower in the 30 HSE group. However, initially, in the CE group, the relative content of DNMT1 was 20% (p < 0.05) higher than that in the control group. Nevertheless, the level in the 30 HSE + 12 h group was not different from the level in the control group, and in the 30 HS + 12 h group, the level was 26% (p < 0.05) lower than that in the control group. At the same time, the relative mRNA levels were correlated with the changes in relative protein expression levels; compared to the level in the control group, the level in the 30 HS group was 57% (p < 0.05) lower, the level in the 30 HS + 12 h group was 61% (p < 0.05) lower and the level in the CE group was 84% (p < 0.05) lower.

The dynamics of the change in the protein expression levels of *de novo* DNMT3a methylase (Fig. 3A) were similar; in the groups 30 HS, 30 HS + 12 h, and 30 HSE, the level was below the control level by 15% (p < 0.05), 16% (p < 0.05), and 14% (p < 0.05), respectively. However, changes in the relative mRNA level were different; in the 30 HS group, no changes were observed; in the 30 HS + 12 h group, the level was 52% (p < 0.05) lower than the level in the control group; and in the CE and 30 HSE groups, the levels were higher than that in the control group by 107% (p < 0.05) and 94% (p < 0.05), respectively.

Like methylases, the protein and mRNA levels of demethylases (Fig. 3D) generally tended to decrease compared to the levels in the control group. Thus, the relative TET1 protein/mRNA levels were 48%/63% lower in the 30 HS group than in the C group (p < 0.05), 54%/53% lower in the 30 HS + 12 h group than in the C group (p < 0.05), 38%/36% lower in the 30 HSE group than in the C group (p < 0.05), and 23% lower in the 30 HSE + 12 h group than in the C group (p < 0.05), while the mRNA levels did not differ from that of the control. Similar results were observed for TET2. The relative protein/mRNA levels in the 30 HS group were 23%/36% lower than those in the C group (p < 0.05). In the 30 HS + 12 h group, the relative protein level was 39% (p < 0.05) lower than that in the control group, while there was no difference in the mRNA levels. In the 30 HSE group, the protein/mRNA levels were 21%/29% (p < 0.05) lower than those in the control group, while in the 30 HSE + 12 h group, no changes were observed.

**Figure 4:**
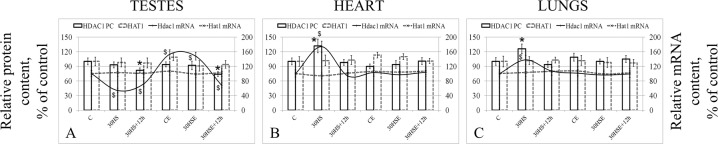
Relative acetylase/deacetylase content protein and mRNA levels. *p < 0.05 in comparison with group C, was used for significant differences in the relative protein content; ^$^p < 0.05 in comparison with group C, was used for significant differences in the mRNA relative content. The left hand y-axis shows the relative protein level (acetylase HAT1 and deacetylase HDAC1) and was used for the histogram columns, while the right hand y-axis shows the relative mRNA content and was used for the line graph. (**A**) testes, (**B**) heart, (**C**) lungs.

There were no differences in the relative protein level of HAT1 histone acetylase (Fig. 4A) or the relative mRNA level of the encoding gene.

The level of deacetylase HDAC1 (Fig. 4A) was 18% (p < 0.05) lower in the 30 HS + 12 h group and 26% (p < 0.05) lower in the 30 HSE + 12 h than in the control group. The relative *Hdac1* mRNA levels in the 30 HS and 30 HSE groups were 46% (p < 0.05) and 28% (p < 0.05) lower than that in the control group, respectively. The use of essential phospholipids led to 53% (p < 0.05) higher *Hdac1* mRNA levels in the CE group and 48% (p < 0.05) higher levels in the 30 HSE group than in the C group. However, the *Hdac1* mRNA level was 32% lower in the 30 HSE + 12 h group than in the C group (p < 0.05).

#### Heart

In the heart tissue, we only managed to evaluate the relative level of the S-phase methylase DNMT1 (Fig. 3B). In the 30 HS group, the protein expression level of DNMT1 was 27% (p < 0.05) lower and the mRNA level was 32% (p < 0.05) lower than those in the control group, while the levels after the 12-hour recovery period (30 HS + 12 h group) did not differ from those of the control. Similarly, the 30 HSE group had protein and mRNA levels that were 20% (p < 0.05), and 27% (p < 0.05) lower than those in group C. At the same time, in the CE and 30 HSE + 12 h groups, the DNMT1 protein expression levels were 52% (p < 0.05) and 24% (p < 0.05) higher, respectively, than that in the control group, with no corresponding changes in the relative mRNA content.

We were unable to evaluate the protein expression level of the DNMT3a *de novo* methylase protein in the heart tissue, although a low level of mRNA was observed, which did not differ among all the study groups.

The relative protein and mRNA levels of TET1 (Fig. 3E) in the 30 HS group were lower than those in the control group by 44% (p < 0.05) and 32% (p < 0.05), respectively. After 12 hours of recovery (in the 30 HS + 12 h group), the protein level was lower than that in the C group by 40% (p < 0.05), while the mRNA level did not differ from that in the control group.

As with DNMT3a *de novo* methylase, we were unable to evaluate the protein expression level of TET2 (Fig. 3E). Nevertheless, the relative mRNA level in the 30 HS group was 29% (p < 0.05) lower than that in the control.

As in the testis tissue, the relative protein expression level of HAT1 histone acetylase (Fig. 4B) and the mRNA level of the corresponding gene did not differ among all study groups. At the same time, the relative protein expression level of deacetylase HDAC1 was 32% higher in the 30 HS group (p < 0.05) than in the C group. At the same time, the *Hdac1* mRNA level was 74% (p < 0.05) higher in the 30 HS group than in the control group. No such changes were noted in the group administered essential phospholipids.

#### Lungs

The changes in the levels of methylases/demethylases and histone acetylase/deacetylase observed in the lung tissue were similar to those in the heart tissue.

The relative protein expression level of DNMT1 (Fig. 3C) and the corresponding mRNA levels were 29% (p < 0.05) and 36% (p < 0.05) lower, respectively, in the 30 HS group than in the control group, while the protein and mRNA levels in the 30 HS + 12 h group were not different from those in the control. In the group administered essential phospholipids, the group that experienced suspension (30 HSE) also had lower levels of the DNMT1 protein and the corresponding mRNA (compared to the CE group), but those levels did not differ from the levels in group C. However, in the CE and 30 HSE + 12 h groups, the DNMT1 protein level was higher than that in group C by 76% (p < 0.05) and 29% (p < 0.05), respectively, while the mRNA levels were higher by 96% (p < 0.05) and 43% (p < 0.05), respectively.

Just as in the heart tissue, we were unable to evaluate the DNMT3a protein content, but the mRNA level, although very low, was the same in all study groups.

The expression of TET1 (Fig. 3F) was not different among groups C, 30 HS, and 30 HS + 12 h. However, in groups administered essential phospholipids, *i.e*., the CE and 30 HSE groups, the relative protein levels exceeded that in the C group by 27% (p < 0.05) and 37% (p < 0.05), respectively, and the relative mRNA levels exceeded that in the C group by 31% (p < 0.05) and 45% (p < 0.05), respectively.

Unlike in the heart tissue, we were able to determine the TET2 protein expression level in the lung tissue. The protein and mRNA levels were 29% (p < 0.05) and 36% (p < 0.05) lower in the 30 HS group than in the control group, respectively. In the same manner, despite the use of essential phospholipids, similar changes occurred in the 30 HSE group; the protein level was lower than the control level by 21% (p < 0.05) and the mRNA level was lower by 25% (p < 0.05).

In the lung tissue, the changes in acetylase/deacetylase levels (Fig. 4C) were identical to those in the heart tissue; there were no changes in the relative proteins levels of HAT1 and the corresponding mRNA levels, while the relative protein expression level of HDAC1 was 26% (p < 0.05) higher in the 30 HS group than in the control group, while the corresponding mRNA level was 38% (p < 0.05) in the 30 HS group than in the control group.

## Discussion

The nature of negative changes arising in the conditions of weightlessness is associated with, among other processes, a whole series of events that take place at the cellular-molecular level. However, to date, the mechanisms underlying cellular perception and transduction of changes in the external mechanical field remain unclear. Thus, the effects of the changing expression of genes, in particular those encoding cytoskeletal proteins, have not yet been explained. In this study, we tested the hypothesis that a change in the expression of genes encoding cytoskeletal proteins may be associated with a change of the methylation status CpG islands in their promoter regions and estimated the content of proteins that regulate the level of methylation.

In previous studies, the relative mRNA levels of a number of genes encoding cytoskeletal proteins were evaluated, and in skeletal muscles, heart, and lungs, decreases in mRNA levels were noted under conditions of prolonged microgravity simulation^8,9,14,15^. At the same time, the use of phospholipids, particularly lecithin, prevented such changes^8^, apparently as a result of changes in the stiffness of the cortical cytoskeleton^19^.

The results obtained in this study, as expected, indicate that in the heart and lungs, there was a decrease in the expression levels of some cytoskeletal genes (*Actn4*, *Des* for heart and *Actn4*, *Des*, *Actb* for lungs) after 30 days of microgravity suspension, and those changes were prevented by the administration of essential phospholipids. Moreover, most of the changes returned to normal levels after a 12-hour recovery period. In contrast, in the testes, there was an increase in expression levels (for *Actn1*, *Actn4*, *Tubb2b*) predominantly after the 12-hour recovery period. One would assume that such changes in the testes were due to emerging cryptorchidism as a result of antiorthostatic suspension. But, Tash *et al*.^20^ had shown that after 6 weeks of male rats suspension there were no changes in the hormonal status, which usually observed under cryptorchidism. On the other hand, our previous data^21^ on spermogram of mice after 30-day suspension indicate that the number of mature sperm cells has decreased. However, the use of essential phospholipids in the same suspension model prevented a decrease of the number of mature forms. Moreover, in both cases, the motility of mature forms was maintained. That is why we suggest that the cryptorchidism was not the cause of the observed changes in the testes tissue.

Thus, we suggest that prolonged microgravity simulation results in adaptation as cells do not need well developed cytoskeleton during long-term mechanical unloading. Therefore, cells experience increased external mechanical load following the return to normal gravity. It requires better developed cytoskeleton, and leads to changes of gene expression as first step. The same effects have been demonstrated in two space experiments^9,22^. No changes were found for animals euthanized in the space (Rodent Research experiment)^9^. However, 12-hours recovery period on the Earth (after BION-M1 mission) resulted in significant changes of cytoskeletal gene expression^22^.

Moreover, the administration of essential phospholipids prevented the development of changes in the early period of readaptation, but led to the increase in the basal expression level (in the CE group) of the genes encoding alpha-actinin-1 and alpha-actinin-4.

Therefore, we decided to attempt to establish the cause of such changes in the expression levels of the genes encoding some components of microfilaments (beta- and gamma-actin and alpha-actinin 1 and 4), microtubules (beta-tubulin), and intermediate filaments (desmin) in different types of cells in mice. These changes may be associated with chromatin remodeling and modifications, changes in genome methylation and others.

In this study genome methylation was analyzed. The main hypothesis was based on the fact that in mammals, one of the main factors controlling gene expression is the methylation of CpG islands in the promoter regions^12–14^. Although methylation patterns are established during development, environmental conditions may lead to changes. Thus, it was shown that, in young rats receiving different levels of maternal care, there are differences in the methylation pattern in the promoter region of the gene encoding the glucocorticoid receptor and that these differences remain stable up to adulthood^23^. Therefore, in this study we focused on comparison of two groups of organs: (1) heart and lungs, and (2) heart and testes. Heart and lungs represent tissues from different germ layers (mesoderm and endoderm respectively) located close to each other in the axis of the body and exposed to the same fluid shift and changes of mechanical load. Heart and lungs are both mesodermal organs experiencing increase and decrease of mechanical stress during rodent suspension. Interestingly, for majority of estimated cytoskeletal genes, the methylation level in the heart tissue was lower than in the lung tissues. It can be related to the contractility of cardiomyocytes. At the same time, level of CpG-island methylation was similar for heart and testes.

A spot analysis of the methylation level of CpG islands in the promoter regions of the studied genes indicated that in the testes, the increases in the relative mRNA levels were correlated with decreases in the methylation of the CpG islands in the promotors. Additionally, in the 30 HS group, the decreases in the relative mRNA levels of *Actn4* and *Des* in the heart and *Actb*, *Actn4* and *Des* in the lungs also correlated with increases in the methylation levels of the CpG islands in their promoter regions. An exception was *Des* in cardiac tissue; the methylation level after the 12-hour recovery period was comparable to that in the control group, while the relative mRNA level was lower than that in the control group and that in the 30 HS group. The lack of correlation between the mRNA and methylation levels in this case may be because the methylation level can determine the efficiency of mRNA synthesis. However, mRNA degradation processes may contribute to the relative content of mRNA, which was estimated by RT-PCR.

All changes of CpG-islands methylation during suspension were prevented by essential phospholipids administration into cardiac and lung, but not testes tissues. By the one hand, there were no differences between control, suspension and recovery groups with phospholipids. On the other hand, phospholipids administration lead to the decrease of CpG-islands methylation level and increase mRNA content for actin-binding proteins (*Actn1* and *Actn4*). And similar situation we observed for skeletal muscle cells^8^. Nevertheless, it should be taken into consideration that testes represent a very heterogeneous tissue, and different cell types contribute to this effect. Moreover, sperm cells and Leydig cells have dramatically different phospholipids composition in their membranes. Therefore, further studies are required to find out which cell type makes a decisive contribution to the observed effect.

Based on the data obtained, we estimated the level of methylation of the genome as a whole. Restriction analysis data indicated that in the testes, the total methylation level and the level of methylation of the CpG islands in the promoters of cytoskeletal genes decrease after 30 days of suspension and remain at the same decreased level after 12 hours of recovery. At the same time, in the heart and lungs, the levels of methylation of the total genome and the CpG islands increased after 30 days of suspension, but returned to the control level after 12 hours of recovery. Such changes in the methylation of the whole genome may be explained by changes in the levels of methylases and demethylases, since the latter is able to change the level of 5 hmC^18,24^. Therefore, we evaluated the level of 5 hmC, but we found that the level did not differ among the studied tissues in any of the groups. Therefore, we further evaluated the relative protein and mRNA levels of the S-phase methylase DNMT1, the *de novo* methylase DNMT3a and the TET family demethylases.

Thus, relative to the level in the control, the level of DNMT1 was lower after 30 days of suspension in all the tissues studied. It should be noted that tissue-specificity of expression was observed; in the heart and lungs, we could not evaluate the level of DNMT3a, which was lower in the testes after 30 days of suspension, nor could we evaluate DNMT1. Moreover, the levels of the TET family demethylases and methylases were generally lower after suspension compared to the control, although tissue specificity was observed in this case as well; all TET proteins, TET1, 2, 3, were measured in the testes; TET1 was measured in the heart; and TET1 and 2 were measured in the lungs. This versatility of TET1 expression may be important for the regulation of the methylation of CpG islands in promoter regions since it predominantly binds specifically to these regions^25^.

Recently, it has been convincingly shown that proteins of the TET family interact with DNA in complex with DNMT1 methylase and that TET2 can also form a complex with the *de novo* methylase DNMT3b^26^. This bond seems to explain the unidirectional change in the levels of methylases and demethylases that took place in our experiment.

Thus, in the testes, the methylation level fell, while in the heart and lungs, the methylation level increased. At the same time, the levels of methylases and demethylases were lower in all the tissues after 30 days of suspension compared with the control. But it should be mentioned, that total methylation level did not change in heart and lung tissues in the groups with phospholipids administration, but methylase/demethylase contents had shown highly variable adaptation pattern. Consequently, it may be assumed that the changes in the level of methylation are associated not with the number of enzymes but with the effectiveness of their recruitment to the genome.

TET acetylation increases the demethylation activity, while deacetylation with HDAC1/2 proteins completely inhibits the activity in complex with DNMT1, leading to the establishment of a hypermethylated state with no changes in the methylase content^26^. For this reason, we evaluated the level of HDAC1 in the tissues of the heart, lungs, and testes.

Really, in the heart and lungs tissues, the establishment of a hypermethylated state after 30 days of antiorthostatic suspension correlated with the increase of the HDAC1 protein level, apparently due to the increase of the its gene expression (since the mRNA content also increased).

There was more difficult situation in the testes. The suspension decreased the total methylation level which was not restored to the control level after 12-hours of recovery. It is possible that hypomethylation in the testes is not related to HDAC1 as its protein level was unchanged after suspension. However, it should be taken into consideration, that HDAC1 level was decreased after 12-hour recovery. It correlated with increased CpG methylation in the promoter regions of cytoskeletal genes in the testes tissue. Thus, HDAC1 can possibly affect local methylation of promoter regions, but it requires future investigations.

On the other hand, the activity of HDAC deacetylase, in the absence of changes in acetylase content (constant HAT1 content was observed in all the tissues studied), may lead to the deacetylation of histones and the establishment of a chromatin state associated with transcriptional silencing. This is because histone acetylation can regulate the transcription efficiency both directly, by reducing the binding of histones to DNA due to the change in the charge^27^, and indirectly, by triggering a long chain reaction, which may involve chromatin remodeling proteins, transcription activators, and proteins that bind methylated DNA and recruit methylases^28–30^.

## Conclusion

In summary, it may be concluded that in the heart and lungs, after 30 days of modeling microgravity the level of methylation of CpG islands in the promoter regions of genes encoding cytoskeletal proteins increases, as does the total level of genome methylation in the absence of changes in the 5 hmC content. However, after 12 hours of recovery, almost all parameters did not differ from the control level. On the contrary, in the testes, the methylation of CpG islands did not change after 30 days of modeling microgravity, but decreased after 12 hours of readaptation. At the same time, events of change methylase/demethylase content were events of decrease content in heart, lungs and testes despite the difference of methylation status.

Comparing these results, we assumed that the changes of the methylation level are associated not with the number of enzymes but with the effectiveness of their recruitment to the genome, which is determined by, among others, HDAC deacetylase. Indeed, the HDAC level was higher in the heart and lungs, which may help to establish the observed hypermethylated state. But, in the testes, the establishment of the hypomethylated state occurred prior to the subsequent changes in the HDAC1 level, suggesting the existence of feedback-type regulation wherein a decrease in the methylation level leads to the decrease in the HDAC level to maintain the hypomethylated status. However, this is only one of the possible explanations that requires further research. In addition, it is necessary to investigate the regulation of expression of genes encoding methylases/demethylases and acetylases/deacetylases under microgravity conditions in different cell types.

Solving these questions can be useful when searching for an answer to the question of signaling pathways that lead to a change in the expression of various genes under microgravity conditions. The latter, in turn, will allow to further understand the nature of cellular mechanosensitivity and develop effective measures to maintain human health during the deep space exploration.

## Materials and Methods

### Experimental design

Microgravity effects were simulated using the Ilyin-Novikov standard model of antiortostatic rodent suspension modified by Morey-Holton^31^. According to this method, animals were suspended by the tail, pretreated with an aseptic solution, so that the hind limbs do not touch the ground, which ensures their unloading. The body of the animal was located at an angle of 30 degrees to the cage floor, while the forelimbs of the animal rested on the surface. The method is widely used to simulate the effects of microgravity in the muscle system (due to unloading of the hind limbs), the cardiovascular system (due to the fluid shift in the cranial direction), and also in the nervous and immune systems^32^.

During the experiment, the animals were kept in standard vivarium conditions and administered standard rodent food and water *ad libitum*. Ten days before the start of the experiment, the animals were randomized into two groups (n = 21 in each); the subjects of one group received essential phospholipids *per os* in the form of EssentsialeR Forte N (A. NATTERMANN and Cie. GmbH, Germany) at a dosage of 500 mg/kg/day throughout the subsequent study period (30 days).

The animals were then further randomly assigned to one of the following groups: C (n = 7), control group; 30 HS (n = 7), the 30-day suspension group; 30 HS + 12 h (n = 7), the group undergoing 30-day suspension followed by 12 hours of recovery; CE (n = 7), control group + essential phospholipids; 30 HSE (n = 7), 30-day suspension group + essential phospholipids; 30 HSE + 12 h (n = 7), the group undergoing 30-day suspension followed by 12 hours of recovery + essential phospholipids.

At the end of the study, after euthanizing the animals, the testes, hearts, and lungs were isolated, weighed, and immediately frozen for subsequent isolation of nucleic acids and proteins. All the procedures conducted with animals were approved by the Commission on Biomedical Ethics of the Institute of Biomedical Problems (IBMP), the State Scientific Center of the Russian Federation and the Federal State Budgetary Institution of Science (Minutes No. 425 dated June 20, 2016).

All experiments were performed in accordance with relevant guidelines and regulations.

### Evaluation of the relative mRNA level of genes encoding cytoskeletal proteins using quantitative PCR

Total RNA from the frozen tissues was isolated using RNeasy Micro Kit (Qiagen, #74004), according to the manufacturer’s instructions. The reverse transcription was performed using d(T)_15_ as a primer with 500 ng of RNA. Estimation of the relative mRNA levels of the investigated genes was performed via real-time PCR with primers selected by Primer3Plus (Table 2), and the results were processed using the 2(-DeltaDeltaC (T) method^33^.

**Table 2:** Primer sequences and product sizes.

Gene	Primer sequence, forward/reverse (5′…3′)	Product size, bp
*Actb* (beta-actin)	*gctgcgttttacaccctttc*/*gtttgctccaaccaactgct*	218
*Actg* (gamma-actin)	*ctggtggatctctgtgagca*/*tcaggagggaagaaaccaga*	184
*Actn1* (alpha-actinin 1)	*ggtcagcagcaacctcctc*/*tctttctccaccttctctcca*	167
*Actn4* (alpha-actinin 4)	*accctgaacagactcccttg*/*gatcgacaagcctccatctc*	168
*Tubb2b* (beta-tubulin 2B)	*ggcagcaagaagctaacgag*/*cgaacacgaagttgtctggc*	302
*Des* (desmin)	*gtgaagatggccttggatgt*/*cgggtctcaatggtcttgat*	182
*Dnmt1* (S-phase methylation)	*ccggaaactcacttggacga*/*tttggcagctggatctctgg*	90
*Dnmt3A* (*de novo* methylation)	*agagcgctttgactccacat*/*ggaccaggaaaaacaaacga*	150
*Tet1* (cytosine demethylase)	*gtgtgggtcgatggctctat*/*cttattcccaccaccgctaa*	208
*Tet2* (cytosine demethylase)	*gttctcaacgagcaggaagg*/*tgagatgcggtactctgcac*	185
*Hat1* (histone aminotransferase 1)	*agagtgccgtggagaagaaa*/*tttcatcatccccaaagagc*	150
*Hdac1* (histone deacetylase 1,2,3,4,6,9)	*ccatgaagcctcaccgaat*/*caaacaccggacagtcctca*	226

### Evaluation of the total DNA methylation level and the methylation of CpG islands in the promoter regions of genes through restriction analysis (MspI/HpaII)

To determine the level of methylation, total DNA was isolated from the frozen tissues using a DNA isolation kit (Synthol, #EX-511) based on a phenol/chloroform method, according to the manufacturer’s instructions.

For the analysis of total CpG methylation in 5′-CCGG-3′ loci, the EpiJET Methylation Analysis Kit (MspI/HpaII) (Thermo Scientific, #K1441) was used according to the manufacturer’s instructions. In cases where the internal cytosine in a 5′-CCGG-3′ tetranucleotide is methylated, the activity of Epi HpaII restrictase is blocked, while the activity of Epi MspI restrictase is not. During restriction, 1 μg of genomic DNA as well as 1 μg each of unmethylated plasmid DNA, pUC DNA/SmaI, and fully methylated plasmid DNA, mpUC DNA/SmaI, were used to control the absence of DNA degradation in the buffer for the restriction and efficiency of MspI and HpaII enzymes. Analysis of the restriction results was carried out in a 1% agarose gel with the FastRuler Middle Range DNA Ladder (Thermo Scientific, #SM1113) size marker; the results were processed using Image Lab (Bio-Rad, USA), with the levels normalized to the total level of genomic DNA in the corresponding sample.

To determine the methylation level of CpG islands in the promoter regions of genes encoding cytoskeletal proteins, the DNA obtained after restriction was reprecipitated with the phenol/chloroform method. Then, quantitative PCR was performed with the corresponding primers selected by Primer3Plus (Table 3), and the results were analyzed according to the manufacturer’s instructions included with the EpiJET Methylation Analysis Kit (MspI/HpaII) (Thermo Scientific, #K1441).

**Table 3:** Sequences of and product size resulting from primers for the promoter regions containing CpG islands and the genes encoding cytoskeletal proteins.

Gene	Primer sequence, forward/reverse (5′…3′)	Product size, bp
*Actb* (beta-actin)	*ccatcgccaaaactcttcat*/*gggttttataggacgccaca*	229
*Actg* (gamma-actin)	*acgggactcaccccagat*/*atggcgatctttccaaaagc*	292
*Actn1* (alpha-actinin 1)	*ctgctggggtttgtagcttg*/*ccttaagctgaaccggagtg*	180
*Actn4* (alpha-actinin 4)	*accccgaaacaggttaaagc*/*gcctgcgcagttcacttg*	243
*Tubb2b* (beta-tubulin 2B)	*cccttctcaagtccgagaca*/*ggtgggttcggtcttttatg*	250
*Des* (desmin)	*ggtctcctgtcagctccttg*/*gtagccctcctgacatcacc*	203

### Evaluation of the level of 5 hmC in DNA through the dot-blotting method

The DNA isolated through the phenol/chloroform method was applied to a nitrocellulose membrane after measuring the concentration and providing denaturation (+95 °С for 5 min, then +4 °С for 3 min) at the following three levels: 1 μg, 500 ng, and 200 ng. The DNA was air dried, welded to the membrane by ultraviolet light and incubated in 4% skimmed milk overnight at +4 °C.

Specific antibodies based on rabbit immunoglobulins (Abcam, #ab214728) at a concentration of 0.5 μg/ml were used to determine the level of 5 hmC according to the manufacturer’s instructions. Biotinylated goat anti-rabbit IgG antibodies (Jackson ImmunoResearch Lab., Inc., #111-035-003) were used as the secondary antibodies at a dilution of 1:10,000. The membranes were then treated with streptavidin solution conjugated with horseradish peroxidase (Sigma, #E2886) at a dilution of 1:10,000. The dots were revealed using 3,3′-diaminobenzidine (Amresco, #E733-50). ImageJ was used to analyze the data obtained.

### Evaluation of protein content by western blotting

Isolation of total protein from the frozen tissues and denaturing electrophoresis were performed according to the Lamely method using the Bio-Rad system (USA). The protein concentration was measured in each sample, and equal amounts of protein were applied to each well. Transfer to the nitrocellulose membrane was carried out according to Towbin *et al*.^34,35^.

To evaluate each protein, specific antibodies based on rabbit immunoglobulins were used at the following concentrations recommended by the manufacturer: 1:1000 for S-phase methylation of Dnmt1 (Abcam, #ab188453); 1:2000 for *de novo* Dnmt3a methylase (Abcam, #ab188470); 2 μg/ml for T-methylcytosine hydroxylase (demethylase) TET1 (Abcam, #ab191698); 1:1000 for TET2 (Abcam, #ab124297); 1:1000 for histone acetylase KAT1/HAT1 (Abcam, #ab194296); and 1:1000 for HDAC1 deacetylase (Abcam, #ab109411). Biotinylated goat anti-rabbit IgG antibodies (Jackson ImmunoResearch Lab., Inc., #111-035-003) were used as secondary antibodies at a dilution of 1:10,000. The membranes were then treated with streptavidin solution conjugated with horseradish peroxidase (Sigma, #E2886) at a dilution of 1:10,000. The protein bands were revealed using 3,3′-diaminobenzidine (Amresco, #E733-50), and the data were analyzed in the ImageJ program.

### Statistical analysis

The results obtained were statistically analyzed with ANOVA, using the post hoc t-test with a significance level p < 0.05 to assess the reliability of differences between the groups. The data are presented as the mean ± standard error of the mean (M ± SE).

## Data Availability

All data generated or analyzed during this study are included in this article.
